# General Dental Practitioners’ Concept towards Using Radiography and Apex-Locators in Endodontics 

**Published:** 2014-10-07

**Authors:** Maryam Raoof, Maryam Heidaripour, Arash Shahravan, Jahangir Haghani, Arash Afkham, Mahsa Razifar, Sakineh Mohammadizadeh

**Affiliations:** a *Oral and Dental Diseases Research Center, Kerman University of Medical Sciences, Kerman, Iran;*; b *Neuroscience Research Center, Institute of Neuropharmacology, Kerman University of Medical Sciences, Kerman, Iran;*; c *New York University, College of Dentistry, New York, USA;*; d *Instructor, Kerman University of Medical Sciences, Kerman, Iran*

**Keywords:** Apex Locator, Dental Radiography, Dentist, Root Canal Therapy, Working Length

## Abstract

**Introduction:** Despite being the gold standard as well as a routine technique in endodontics, radiographic working length (WL) determination owns many drawbacks. Electronic apex-locators (EALs) are recommended to complement radiographies. The aim of this study was to evaluate the perceptions of Iranian general dental practitioners (GDPs) towards using radiography and EAL. **Methods and Materials:** Three hundred and ninety one GDPs attending the 53^th^ Iranian Dental Association Congress completed a questionnaire focusing on the use of radiography and EALs during the various stages of root canal treatment. The data was analyzed with the chi-square test with the level of significance set at 0.05. The results were then calculated as frequencies and percentages. **Results:** More than half of the GDPs reported using radiographs as the sole method for WL determination. A total of 30.4% of the practitioners were using the combined approach during root canal therapy of a single-rooted tooth, while 38.9% used this method in multi-rooted teeth. Approximately half of the respondents would not order follow-up radiographies after root canal treatment. **Conclusion: **Radiography continues to be the most common method for WL determination in Iran.

## Introduction

Root canal treatment (RCT) is considered an essential component of dental care; however, it is still a challenging procedure for general dental practitioners (GDPs) [1]. Studies have shown that more than 50% of teeth do not receive proper endodontic treatment and approximately 30-50% of root canal treated teeth have radiographic signs of apical periodontitis [2].

One of the main difficulties during endodontic treatment is identifying and maintaining the biological length of the root canal system. The accurate working length (WL) determination is a crucial factor that influences the outcome of RCT [3, 4]. Several methods have been used to determine the WL of root canals.

Radiographic method is traditionally the most common technique in WL determination [5]. Moreover, it is the essential component of all stages of RCT from diagnosis and treatment planning to mechanistic stages of treatment and assessment of endodontic results [6]. However, a number of disadvantages make this technique unsuitable for every situation. Radiography produces a two dimensional image of the roots [7]. Furthermore, superimposition of other structures usually makes WL determination difficult. Tooth inclination and angulation of the x-ray tube also have an influence on the results. Other disadvantages include technique sensitivity, subjectivity [5] and the danger of ionizing radiation [8].

The development and production of electronic apex locators (EALs) for locating the canal terminus, has been one of the most remarkable innovations in endodontics that has simplified and shortened the treatment procedure and consequently has improved its outcome [9]. 

**Table 1:** Demographic information of the participants (*n=*391)

**Characteristic**		**N (%)**
**Age**	**≤35**	173 (44.2)
	**36-45**	130 (33.2)
	**>46**	88 (22.5)
**Gender**	**Male**	205 (52.4)
	**Female**	186 (47.6)
**Year of graduation**	**Before 1996**	108 (27.6)
	**1997-2006**	130 (33.2)
	**After 2006**	153 (39.2)
**Practice experience**	**≤5**	132 (33.8)
	**6-10**	73 (18.7)
	**11-15**	51 (13.0)
	**16-20**	67 (17.1)
	**>20**	68 (17.4)
**Working place**	**Private office**	185 (47.3)
	**Clinic**	95 (24.3)
	**Both **	111 (28.4)

Nevertheless, a number of researchers have stressed the benefits of combining both radiographic and electronic methods to optimize measurement accuracy [11]. In this regard, some investigations have shown reluctance among clinicians to use EALs [16-18].

To date no national study has been performed in this regard and the aim of this research was to investigate the concept of using radiography and EALs during RCT by GDPs who participated in the 53^th^ congress of Iranian Dental Association.

## Methods and Materials

This cross-sectional study was approved by the Research Ethics Committee at Kerman University of Medical Sciences (Grant no.: K-92-224). The questionnaire used in this research was adapted from a previous study with only a few modifications [16]. The questionnaire included demographic information (age, gender, the year of graduation, *etc*.) and some questions about the use of radiography and EALs (from any generation) amongst GDPs during the various stages of endodontic treatment.

To estimate the content validity index (CVI), six endodontists commented on each question. The CVI of each question was in the range of 0.8 to 1, which confirmed the validity of the questionnaire. A pilot study on 20 dentists was conducted. The participants acquired 35% of total score. In this regard, the sample size with *α*=0.05 and *d*=0.04 was calculated as 350. To determine the reliability of the questionnaire, a test-retest method was used. After 10 days, the subjects completed the questionnaire again. The reliability for each question showed that the questionnaire had kappa index more than 0.60 indicating an acceptable reliability limit.

The questionnaire was personally distributed among 430 randomly selected GDPs who participated in the 53^th^ Congress of Iranian Dental Association in Tehran.

All participants were given an explanation regarding the objective and potential benefit of the study and they were ensured of the confidentiality of information provided. A total of 391 questionnaires were returned.

The collected data was entered into SPSS 15 software (SPSS, Chicago, IL, USA) for windows and was analyzed using the chi-square test with the level of significance set at 0.05. The results were then calculated as frequencies and percentages.

## Results

Among 430 distributed questionnaires, a total of 391 (90.93%) papers were completed and returned by the participants. Minimum and maximum age of participants was 24 and 77 years old, respectively (mean age of 38.65 years). Totally, 52.4% of the respondents were male, and 47.6% were female. The demographic information of the participants is presented in Table 1.

When assessing the use of the pre-operative radiograph, more than 53% of practitioners indicated that before RCT, they always take a pre-operative radiograph. However, only 36.8% of the participants were used to routinely taking the immediate post-operative radiographs to ensure the quality of RCT (Table 2).

Table 3 shows the number of radiographic exposures employed during the RCT of a permanent maxillary first molar (MFM). Totally, 78% of practitioners indicated that they take one pre-operative radiograph, while 23.6% claim using two radiographs for WL determination. Table 4 presents the respondents’ opinions towards the most accurate method for definite WL determination. Among all participants, 69.8% believed that combining both radiographic and electronic methods optimizes measurement accuracy. Table 5 details the use of EAL in RCT of single- and multi-rooted teeth. More than half of the practitioners considered the use of radiography as the only method for establishing WL in both situations. Table 6 summarizes the clinicians’ opinions regarding the apical position of the WL. Almost 80% of practitioners aimed at achieving a WL between 0.5 and 1 mm short of the radiographic apex.

**Table 2:** The frequency of taking pre-operative and post-operative radiographs (*n=*391)

**Use of radiographs**	**Always N (%)**		**Most cases N(%)**	**Occasionally N (%)**	**Almost never N (%)**	**Never N (%)**
**Pre-operative film**		208 (53.2)	116 (29.7)	47 (12.0)	15 (3.8)	5 (1.3)
**Post-operative film**		144 (36.8)	99 (25.3)	89 (22.9)	53 (13.5)	6 (1.5)

**Table 3:** The frequency of taking radiography during the various stages of endodontic therapy of a maxillary first molar (*n*=391)

**Number of radiographs **	**None N (%)**	**One N (%)**	**Two N (%)**	**Three N (%)**	**Four N (%)**
**Pre-operative **	22 (5.7)	305 (78.0)	48 (12.0)	14 (3.7)	2 (0.6)
**Working length **	15 (3.8)	276 (70.6)	92 (23.6)	8 (2)	0 (0)
**Master-cone fit **	92 (23.5)	245 (62.7)	49 (12.5)	5 (1.3)	0 (0)
**Post-operative **	59 (15)	289 (73.9)	35 (9.3)	4 (1.0)	4 (1.0)

**Table 4:** Respondents’ opinion towards the most accurate method for definite working length (WL) determination

**Most accurate method **	**N (%)**
**Radiography**	86 (22.0)
**Apex locator**	21 (5.4)
**Radiography and apex locator**	273 (69.8)
**Use of paper point**	2 (0.5)
**Tactile sense **	9 (2.3)

Participants were asked at what distance they would consider retaking WL measurement radiography when there was a difference between the tip of the file and the root apex. Table 7 details the respondents’ responses. More than 40% reported that they would retake the radiography when the distance was 2.1-3 mm from the radiographic apex.

Based on using the various radiographic techniques, only 32.2% of the GDPs utilize the parallel technique, while 67.8% applied the bisecting angle technique. Table 8 outlines the responses of GDPs’ to follow-up appointment after RCT. Only 19.9% of the participants believed that RCT needs followed-up radiographies. Table 9 illustrates the time length (months) required for ordering follow-up radiographs after RCT.

Although most of the items were unrelated to age, younger practitioners took immediate post-operative radiographs more commonly than their older colleagues (*P*=0.01). Conversely, the paralleling radiographic technique was more popular amongst the older practitioners (*P*=0.01). Also, older clinicians were more likely to hold the belief that all patients should be followed-up (*P*=0.0001).

There was an association between the follow-up visits and the year of graduation (*P*=0.003). Recent graduates were less likely to follow-up all patients. No significant relationship was found between the year of graduation and other variables.

There was an association between practicing location with taking pre-operative and post-operative radiographies and number of radiographies for initial WL determination of a MFM (*P*<0.05). The dentists who worked in clinics responded more correctly to questions.

Also, there was a significant relationship between practice experience and number of radiographies taken to determine the initial WL in treatment of a MFM (*P*=0.02) and the follow-up visits (*P*=0.0001). The proportion of participants who considered the follow-up visits essential for all vital and necrotic teeth, was higher among dentists with more than 20 years of professional experience.

## Discussion

The present study evaluated the use of radiography and EALs during RCT by GDPs because epidemiological studies suggest that the failure rate is distinctly higher for teeth treated by non-specialist dentists [19]. Moreover, it seems that GDPs provide the majority of dental treatments in Iran.

In questionnaire-based studies, controversy exists with regard to the minimum level of the response rate which ensures the absence of non-response bias. Nevertheless, a range of 70-80% has been suggested [19]. In this study, the overall response rate was 90.93% that can be considered satisfactory and may be indicative of a growing interest among the GDPs regarding the topic.

Unfortunately, in our study only 53.2% of the dentists always prescribed the pre-operative radiographs. This figure was considerably lower compared to the data released by Orafi and Rushton [16], Palmer *et al*. [20] and Ravanshad *et al.* [1] who stated that 83.9%, 98.5% and 72% of the participants reported using a preoperative radiography.

It is important to note that the post-operative radiograph provides an important clinical record of the quality of the RCT undertaken and also acts as a baseline for subsequent follow-up radiographies. A rather negative finding in our study was that 36.8% of the clinicians always took post-operative radiographs. This figure was lower than many earlier studies [16, 20-22]. As conducting RCT without pre-operative and post-operative radiographs is below the standard of care [23], it appears that our participants are not complying with endodontic guidelines. Notwithstanding, the present research did reveal a marked improvement from the level recorded in another national study [1], in which only 10% of clinicians routinely reported taking a post-operative radiograph. This could be attributed to the advancement in the undergraduate study and increased professional legal disputes (*i.e. *patient legal complaints) in recent years.

**Table 5:** The frequency of using electric apex locator (EAL) and radiography (RG) or only one of them in single- and multi-rooted teeth

**Method used**	**EAL N (%)**	**EAL and RG N (%)**	**RG N (%)**
**Single-rooted tooth**	38 (9.7)	119 (30.4)	234 (59.8)
**Multi-rooted tooth**	29 (7.4)	152 (38.9)	210 (53.7)

**Table 6:** Respondents beliefs towards the apical limit of canal preparation from the radiographic apex

**Distance (mm)**	**0.5-1**	**1.1-2**	**2.1-3**	**Radiographic apex**
**N (%)**	306 (78.3)	47 (11.9)	7 (1.9)	31 (7.9)

**Table 7:** Limit for radiographic retakes by respondents

**Limits (mm)**	**1-2**	**2.1-3**	**3.1-4**	**4.1-5**	**5<**
**N (%)**	79 (20.2)	168 (43)	113 (28.9)	16 (4.2)	15 (3.7)

The number of radiographs exposed during treatment varied from three to five. A previous study found variability in the numbers of radiographs taken during the RCT of a maxillary molar with 43.3% of clinicians taking three radiographs and 24.6% taking four [24]. Palmer *et al.* [20] also reported similar findings. In the present study, most of the clinicians reported taking four radiographies during treatment of a MFM.

The majority of the practitioners (70.6%) relied on a single radiograph to determine the WL of the roots of the MFM. This is in line with the findings of some other studies in the UK and New Zealand [16, 24].

WL radiographs during treatment of MFMs were used by 96.2% of the participants in the present study which was higher than the number reported by other studies. A considerable variability exists in the use of the WL radiograph ranging from 61% to 89% [20-22, 25].

Adequate cleaning and disinfection is essential for successful RCT. Therefore, correct estimation of the length of the canal is a crucial step in endodontic treatment [3, 4, 26]. Over the years, numerous methods have been advocated to estimate the root canal WL. Traditionally, radiography was universally acknowledged as the best and most common technique in this regard [5]. However, the limitations of this method are well known, including two-dimensional images [7], superimposition of structures and geometric distortion [5]. As the electronic method for WL measurement eliminates some of the problems associated with traditional radiographic methods, it’s accuracy and ease of use has progressed significantly during recent years [10].

In the current study, a noticeable 23.5% of GDPs recorded no use of radiography to determine the master cone position in the canal. The finding may be due to the availability of accurate EALs and the ethical importance of reducing multiple x-ray exposures.

Overall, a small number of participants (2.3%) relied upon tactile sensation when determining the WL. Tactile sensation, although useful in experienced hands, has many limitations. The files may bind against the walls at any position along the canal, or they may perforate apically [27].

In the current survey, over half of the dentists reported using only radiography for WL determination (Table 5). Unfortunately, the result does not show that the armamentarium is being incorporated into modern endodontic practice. More accurate WL determination could be achieved by a combination of conventional radiographic techniques with modern EALs [11]. In addition, EALs reduce the number of radiographs required, and consequently save time and minimize the radiation dose [15]. There seems to be a reluctance to use EALs in some other countries as well. In the UK for example, more than 35% of GDPs reported using EALs to determine the WL in single- and multi-rooted teeth [16]. Bjorndal and Reit [17], reported that only 23% of Danish dentists used EALs. In a study carried out by Hommez *et al.* [18], 16% of the participants from Belgium used EALs occasionally. Interestingly, Palmer *et al.* [20] found that 57.3% of practitioners in the north west of England use radiographs as the only method for establishing WL.

Nevertheless, a survey of endodontic practice was conducted in 2008 in Iran [1]. The authors reported that 84% of the dentists used radiographies for determining the WL, and only 2.7% used EALs. Results of a second survey, revealed that 45.2% of GDPs used EALs in Iran [9]. The present study also found that about 30.4% of the practitioners were using the combined approach of a WL radiograph and an EAL during RCT of a single-rooted tooth while, 38.9% used this method in multi-rooted teeth. There is an overall increasing trend for utilizing EALs. This may be due to the availability of accurate, user-friendly, and easy to use devices.

The most common response for position of preparation termination point was 0.5 to 1.0 mm short of the radiographic apex. According to Orafi and Rushton [16], 87.7% of the GDPs prepared canals 0.5-1 mm short of the radiographic apex, while Ravanshad *et al.* [1] reported this tendency to be 80%. In contrast, in a Flemish survey, 38.9% of the GDPs instrumented the canals 1 mm short of the radiographic apex independent of the periapical pathosis [28]. The apical end point of the WL is one of the major controversies in RCT. The European concept [23] is to leave the root filling 1-2 mm short of the apex, whilst in North America [29] clinical practice is to shape the canal to the radiographic apex terminus. Moreover, in the classic study by Sjogren *et al.* [30], it was stated that in cases where the pulp was necrotic and infected, the WL should be selected within 1 mm of the radiographic apex. The optimal WL in teeth with vital pulp appears to be 1-2 mm from the radiographic apex [31].

**Table 8:** Frequency of taking pre-operative, post-operative and follow-up radiographies

**Situation**	**N (%)**
**Teeth with necrotic pulp, without considering the existence of apical lesions**	38 (9.8)
**Teeth with necrotic pulp and apical lesions, without considering the size of lesion**	156 (40)
**Teeth with necrotic pulp and large apical lesions**	119 (30.3)
**All vital and necrotic teeth**	78 (19.9)

**Table 9:** Frequency of taking follow-up radiographies after endodontic treatment

**Period (months)**	**No follow-up**	**6 **	**12 **	**18 **	**24 **	**36 **	**48 **	**>48 **
**N (%)**	191 (48.9)	141 (36)	48 (12.3)	5 (1.3)	6 (1.5)	0 (0)	0 (0)	0 (0)

It was noticeable that 43% of respondents reported reproduction of WL radiographs when the difference between the end of the file and the radiographic apex was between 2.1 to 3 mm, while 28.9% claimed to retake the radiography when the distance was 3.1 to 4 mm. In the study by Orafi and Rushton [16], 63.5% of the GDPs took another radiograph when the difference between the file tip and the radiographic apex was between 3 and 5 mm.

For endodontic purposes, the paralleling technique produces the most accurate periradicular radiograph. It provides images with the least dimensional distortion, minimal superimposition, and increased clarity. Although the bisecting angle technique is still utilized by some practitioners, it’s not the method of choice for endodontic purposes. The bisecting-angle technique causes noticeable distortion and makes it difficult for the clinician to reproduce radiographs at similar angulations to assess healing in follow-up visits [32, 33]. Unfortunately, in this study only 32.2% of the participants reported using the parallel radiographs which is almost similar to Orafi and Rushton’s [16] study (35%). In New Zealand, 24.6% of clinicians always utilized the parallel radiographs [24]. Presumably, this dilemma may be attributed to the clinicians’ education at undergraduate level. Tugnait *et al.* [34] stated that good radiographic practice was associated with an acquired postgraduate qualification. The paralleling technique and utilization of periapical film holders should be taught as a part of the undergraduate dental curriculum. Voluntary or mandatory continuing education courses are other possible ways to improve the knowledge and skills of GDPs.

For monitoring the outcome of every RCT, clinical and radiographic follow-ups must be addressed at regular intervals for a minimum observation period of 1 year. Although, longer durations may be required where healing is incomplete or there is a history of trauma [35]. It was surprising to find that about 80% of the participants believed that only teeth with pulp necrosis should be followed-up. Moreover, about half of the GDPs did not follow up their patients after RCT. In the study by Orafi and Rushton [16], the most common follow-up period was 1 year. In the study conducted by Chandler and Koshy [24] a significant number of clinicians (13.2%) continued to recall their patients for 4 years. 

## Conclusion

Based on the results of the present study, Iranian general dental practitioners were not following the standards of endodontic treatment. There was reluctance amongst the participants to take post-treatment radiographies, use the paralleling technique of intraoral radiography and adopt electronic apex locators. In addition, a noticeable proportion of participants did not provide follow-ups for their patient after root canal therapy.
